# Exogenous Melatonin Enhances Cold, Salt and Drought Stress Tolerance by Improving Antioxidant Defense in Tea Plant (*Camellia sinensis* (L.) O. Kuntze)

**DOI:** 10.3390/molecules24091826

**Published:** 2019-05-12

**Authors:** Jiahao Li, Yiqing Yang, Kang Sun, Yi Chen, Xuan Chen, Xinghui Li

**Affiliations:** Tea Research Institute, Nanjing Agricultural University, Nanjing 210095, China; 2016104091@njau.edu.cn (J.L.); 14216111@njau.edu.cn (Y.Y.); sunkang@njau.edu.cn (K.S.); 2017804134@njau.edu.cn (Y.C.)

**Keywords:** *Camellia sinensis*, tea plant, melatonin, cold stress, salt stress, drought stress, antioxidant, reactive oxygen species

## Abstract

Melatonin is a biological hormone that plays crucial roles in stress tolerance. In this study, we investigated the effect of exogenous melatonin on abiotic stress in the tea plant. Under cold, salt and drought stress, increasing malondialdehyde levels and decreasing maximum photochemical efficiency of PSII were observed in tea leaves. Meanwhile, the levels of reactive oxygen species (ROS) increased significantly under abiotic stress. Interestingly, pretreatment with melatonin on leaves alleviated ROS burst, decreased malondialdehyde levels and maintain high photosynthetic efficiency. Moreover, 100 μM melatonin-pretreated tea plants showed high levels of glutathione and ascorbic acid and increased the activities of superoxide dismutase, peroxidase, catalase and ascorbate peroxidase under abiotic stress. Notably, melatonin treatments can positively up-regulate the genes (*CsSOD*, *CsPOD*, *CsCAT* and *CsAPX*) expression of antioxidant enzyme biosynthesis. Taken together, our results confirmed that melatonin protects tea plants against abiotic stress-induced damages through detoxifying ROS and regulating antioxidant systems.

## 1. Introduction

The tea (*Camellia sinensis* (L.) O. Kuntze) plant is an important economic crop that has been planted in China for nearly 2000 years [1]. As a perennial evergreen woody crop, tea plants are often subjected to various abiotic stresses such as drought, high salt and low temperature during the growth process [2], which are the main environmental factors that affect the geographical distribution of tea plant and limit the yield of tea [3]. Thus, effective ways to improve the abiotic stress tolerance on tea plants are urgently needed.

Plants grown under environmental stress show the inhibition of growth and serious morphological, metabolic, and physiological anomalies ranging from chlorosis or leaf rolls to lipid peroxidation and protein degradation [4,5,6]. Malondialdehyde (MDA) content is generally considered as an indicator of membrane structural integrity. The level of MDA content has increased significantly under abiotic stress, resulting in decreased membrane fluidity and destroyed ion homeostasis on plants [7]. Photosynthesis, the most important physicochemical process in higher plants, is very sensitive to abiotic stress [8,9]. Environmental stress can significantly affect the content of chlorophyll and the activity of key enzymes in photosynthesis [10]. Furthermore, abiotic stress directly affects the photosynthetic system, mainly by inducing photoinhibition at both photosystem I (PSI) and PSII [11], stress-induced inhibition of the photosynthetic electron transport results in excessive accumulation of toxic reactive oxygen species (ROS) such as hydrogen peroxide (H_2_O_2_) and superoxide anion (O_2_^−^) [10,12]. The excessive accumulation of ROS promotes degradation of chlorophyll and reduces the photochemical efficiency of photosystem II (PSII) forming a vicious cycle [13,14]. Moreover, the excessive ROS produced under abiotic stress is a harmful factor, which causes lipid peroxidation, enzyme inactivation and DNA damage [15]. In response to over-induction of ROS under abiotic stress, plants employ efficient detoxifying networks, including increased activities of antioxidant enzymes, such as superoxide dismutase (SOD), catalase (CAT), peroxidase (POD) and ascorbate peroxidase (APX), and regulated contents of non-enzymatic antioxidants, including glutathione (GSH) and ascorbic acid (ASA) [5,16]. Therefore, it is essential for plants to maintain the content of ROS at an appropriate level to resist abiotic stress.

Melatonin (*N*-acetyl-5-methoxytryptamine) is an indole hormone involved in multiple biological processes [17]. Since the discovery of melatonin in vascular plants in 1995 [18,19], the role of melatonin related to plant physiology has attracted widespread attention. Subsequent studies have proven melatonin plays an important role in the regulation of plant growth and development [20] and defense against abiotic stresses such as extreme temperature, excess copper, salinity, and drought [21,22,23]. According to new findings, melatonin plays several important functions in plants. It may act as a plant growth regulator in rooting, seed germination, and delay in leaf senescence and other morphogenetic features [24,25,26]. Furthermore, melatonin and auxin (IAA) share tryptophan as a precursor for biosynthesis and have similar structural components [26]. However, there is no conclusive data on the metabolic transformation between melatonin and IAA in plants.

Many researchers have demonstrated that melatonin can protect plants against ROS and consequent alleviation of oxidative stress [22,27]. Melatonin itself is an effective antioxidant that directly scavenges ROS. Notably, its metabolite, *N*1-acetyl-*N*2-formyl-5-methoxykynuramine (AMFK), which has stronger antioxidant activity than melatonin, can also directly and efficiently scavenge ROS [20,28,29]. What is more, melatonin also possesses antioxidant activity operating by modulating antioxidant enzymes and enhancing cellular non-enzymatic antioxidants [30,31]. ROS-scavenging enzymes (SOD, POD, CAT and APX) and antioxidants (GSH and ASA) are necessary to provide cells with highly efficient machinery for detoxifying ROS [32,33]. Recently, solid evidence was observed that plants accumulate high levels of melatonin when subjected to extreme environmental conditions [25] and exogenous application of melatonin helps improve tolerance to stresses [34,35]. As a free radical scavenger, melatonin protects the plants from oxidative stress under different environmental stresses in all species [36,37]. However, it is still unclear whether such function of melatonin against abiotic stress is universal for other plant species.

Although remarkable progress has been made in investigating the role of melatonin in responses to abiotic stress, very less information about the effects of melatonin on abiotic stress tolerance in tea plants is available. In the present study, we explored the regulatory mechanisms controlling melatonin-mediated abiotic stress tolerance in tea plants and tried to understand the impact of melatonin on abiotic stress in tea plants. Hence, we analyzed the effects of melatonin on lipid peroxidation, ROS accumulation, antioxidant defense system, and photosynthetic capacity in tea seedling exposed to cold, high salt and drought stresses.

## 2. Results

### 2.1. Effects of Exogenous Melatonin on Photosynthesis in Tea Plants under Abiotic Stress

**Figure 1:**
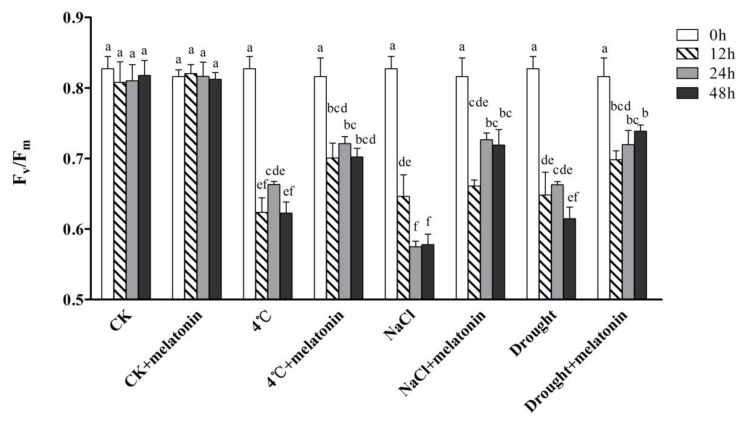
Effect of exogenous melatonin on photochemical efficiency of PSII (Fv/Fm) in tea leaves on the designated times. The tea plants were subjected to control (CK), cold stress (4 °C), high salt (300 mM NaCl) and drought stress (200 g·L^−1^ PEG) with different treatments (CK and 100 μM melatonin). All experiments were carried out at 25 °C except for cold stress (4 °C). Data represent means ± SD of three replicate samples. Different letters indicate significant differences according to Duncan’s multiple range test (*P* < 0.05).

The effect of exogenous melatonin on maximum photochemical efficiency of PSII (Fv/Fm) of tea plants under cold (4 °C), salt (NaCl) and drought stress are shown in Figure 1. After 7-day-melatonin pre-treatment, tea plants treated with melatonin or not showed no significant differences at 0 h (Figure 1). When an abiotic stress was applied, the value of Fv/Fm significantly decreased under cold, salt or drought stress. As a result, the photosynthesis of both melatonin-pretreated and non-treated plants was inhibited. However, almost all melatonin treatments significantly alleviated the deleterious effects of abiotic stress. The application of 100 μM melatonin significantly improved Fv/Fm of tea leaves compared with 4 °C, salt and drought alone treatment. In tea plants treated with 100 μM melatonin, the Fv/Fm value significantly increased by 12.6%, 24.2% and 20.2%, compared to non-melatonin treated plants under 4 °C, NaCl and drought treatments, respectively at 48 h.

### 2.2. Effects of Exogenous Melatonin on MDA Contents and ROS Levels in Tea Plants under Abiotic Stress

**Figure 2:**
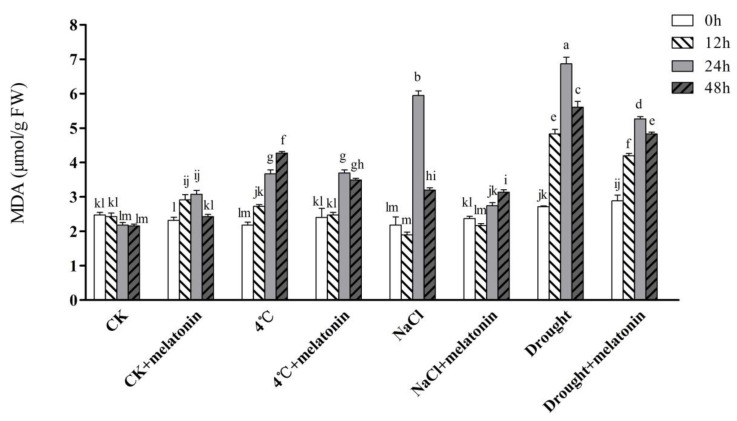
Effect of exogenous melatonin on malondialdehyde (MDA) contents in tea leaves on the designated times. The tea plants were subjected to control (CK), cold stress (4 °C), high salt (300 mM NaCl) and drought stress (200 g·L^−1^ PEG) with different treatments (CK and 100 μM melatonin). Data represent means ± SD of three replicate samples. Different letters indicate significant differences according to Duncan’s multiple range test (*P* < 0.05).

A change in the contents of MDA with melatonin treatment is presented in Figure 2. Obviously, the MDA content, which reflects damage to the cell membrane, was significantly increased by cold, salt and drought stress in tea plants, and these increases were attenuated by pretreatment with melatonin treatments. Compared to CK, NaCl and drought treatments increased the MDA content of tea seedlings by 172.5% and 215.1%, respectively at 24 h (Figure 2). Nevertheless, pretreatment with 100 μM melatonin reduced the MDA content of tea seedlings by 53.9% and 23.3% in comparison with non-melatonin treated tea plants under salt and drought stress, respectively at 24 h. Notably, melatonin-treated plants did not lead to distinct changes both at 48 h of drought and 48 h of NaCl treatment compared with non-melatonin treated plants.

As important indicators of oxidative damage during abiotic stress treatment, H_2_O_2_ and O_2_^−^ contents were assayed among control and melatonin-pre-treated plants (Table 1). Under control conditions, exogenous melatonin showed that there were significant changes in H_2_O_2_ and O_2_^−^ contents. When the 4 °C, NaCl and drought treatment was applied, both H_2_O_2_ and O_2_^−^ levels of the tea leaves increased sharply, and reached a peak at 48 h of NaCl treatment (1.53 μmol/g FW) and 48 h of 4 °C treatment (1.70 μmol/g FW), respectively (Table 1). Strikingly, melatonin application significantly decreased the excess ROS accumulation compared to non-melatonin treated groups under abiotic stress. In addition, compared to CK, exogenous melatonin somewhat suppressed the production of O_2_^−^ at 12 h and 24 h of NaCl treatment. The obtained results indicate that exogenous melatonin might alleviate abiotic stress-triggered ROS accumulation and cell membrane damage in tea plants.

### 2.3. Effects of Exogenous Melatonin on ROS-related Antioxidant Enzymes in Tea Plants Response to Abiotic Stress

**Figure 3:**
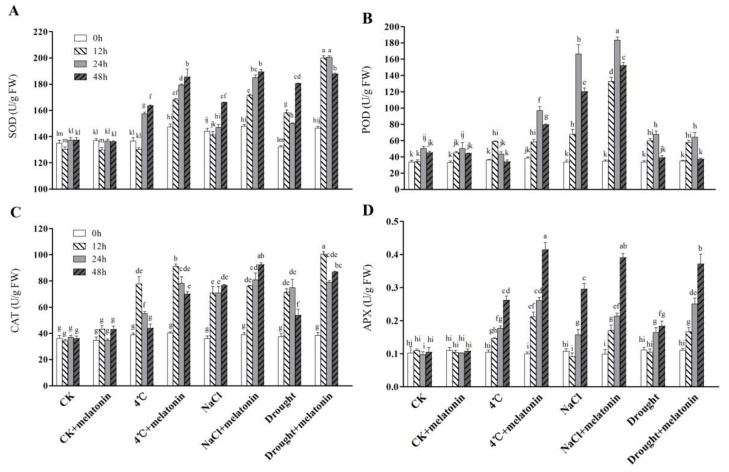
Effect of exogenous melatonin on the activities of antioxidant enzymes in tea leaves on the designated times. (**A**) superoxide dismutase (SOD), (**B**) peroxidase (POD) (**C**) catalase (CAT), (**D**) ascorbate peroxidase (APX). The tea plants were subjected to control (CK), cold stress (4 °C), high salt (300 mM NaCl) and drought stress (200 g·L^−1^ PEG) with different treatments (CK and 100 μM melatonin). Data represent means ± SD of three replicate samples. Different letters indicate significant differences according to Duncan’s multiple range test (*P* < 0.05).

The responses of antioxidant enzymes closely related to ROS were studied in tea plants (Figure 3A–D). Under control conditions (CK), melatonin did not lead to any significant change in the activities of SOD, POD, CAT and APX. When 4 °C, NaCl and drought treatments were performed, the activities of antioxidant enzymes were greatly induced compared with non-stressed tea leaves. In most cases, the exogenous application of melatonin treatments induced the activities of antioxidant enzymes at a higher level in comparison with non-melatonin-treated treatments under the abiotic stress condition. However, the activity of POD had no significant change after melatonin treatment under drought stress (Figure 3B). The results show that exogenous application of melatonin stimulated ROS-related antioxidant enzymes activities in tea plants under abiotic stress.

### 2.4. Effects of Exogenous Melatonin on Non-Enzymatic Antioxidant in Tea Plants Response to Abiotic Stress

**Figure 4:**
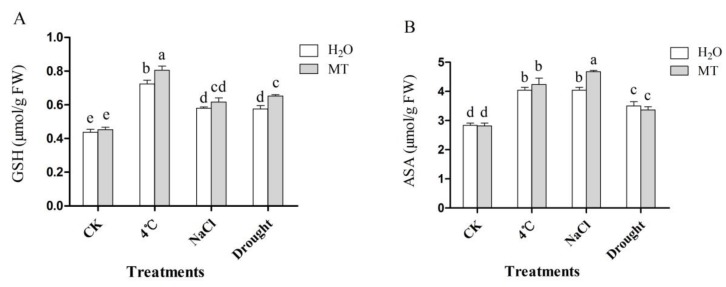
Effect of exogenous melatonin on non-enzymatic antioxidant in tea leaves at 48 h after abiotic stress treatment. (**A**) glutathione (GSH), (**B**) ascorbate acid (ASA). The tea plants were subjected to control (CK), cold stress (4 °C), high salt (300 mM NaCl) and drought stress (200 g·L^−1^ PEG) with different treatments (CK and 100 μM melatonin). Data represent means ± SD of three replicate samples. Different letters indicate significant differences according to Duncan’s multiple range test (*P* < 0.05).

Two important non-enzymatic antioxidants in the ascorbate-glutathione (AsA-GSH) cycle, the contents of GSH and ASA were examined in non-treated and melatonin-pretreated plants under control and abiotic stress conditions. As shown in Figure 4, the GSH content increased when the tea plant was subjected to cold, salt and drought stress. Moreover, treatment with melatonin resulted in a further increase, especially melatonin treatment under 4 °C and drought treatment, were 11.3% and 13.3% higher compared with non-melatonin treated treatment, respectively (Figure 4A). Additionally, stress-treated treatment presented significantly higher contents of ASA in comparison with CK, while melatonin-treated strengthened this trend, except under drought treatment (Figure 4B). These data indicate that exogenous melatonin stimulated non-enzymatic antioxidants under environmental stress.

### 2.5. Effects of Exogenous Melatonin on Gene Expression in Biosynthesis of Antioxidant Enzymes in Tea Plants Response to Abiotic Stress

**Figure 5:**
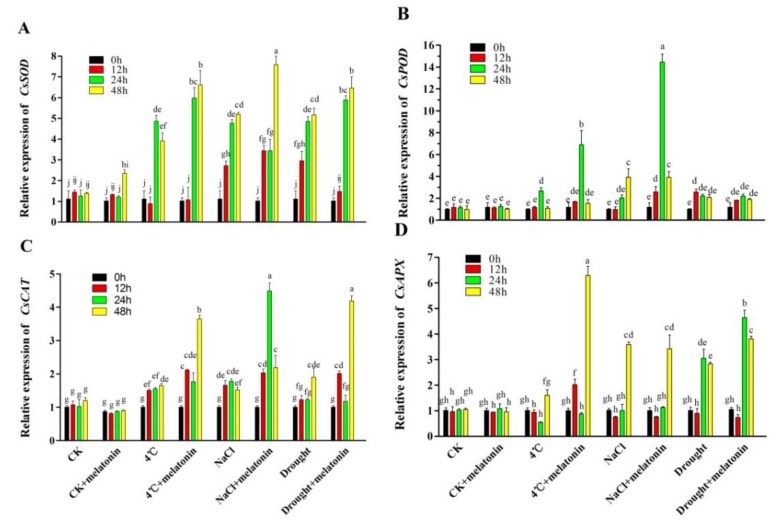
Effect of exogenous melatonin on gene expression in the biosynthesis of antioxidant enzymes in tea leaves on the designated times. (**A**) *CsSOD*, (**B**) *CsPOD* (**C**) *CsCAT*, (**D**) *CsAPX*. The tea plants were subjected to control (CK), cold stress (4 °C), high salt (300 mM NaCl) and drought stress (200 g·L^−1^ PEG) with different treatments (CK and 100 μM melatonin). Data represent means ± SD of three replicate samples. Different letters indicate significant differences according to Duncan’s multiple range test (*P* < 0.05).

To understand the molecular mechanism of melatonin-induced abiotic stress detoxification in tea plants, the expression levels of *CsSOD*, *CsPOD*, *CsCAT* and *CsAPX* were analyzed, which are responsible for the biosynthesis of antioxidant enzymes. Similar to the changes in the activities of antioxidant enzymes, the transcript levels of *CsSOD*, *CsPOD*, *CsCAT* and *CsAPX* were all induced by 4 °C, NaCl and drought treatment (Figure 5). Importantly, melatonin further increased the transcript levels of those genes. On the whole, the expression of *CsSOD* and *CsCAT* was further upregulated in melatonin-pre-treated plants from 12 h compared with non-treated plants (Figure 5A,C). In addition, melatonin-pretreated plants had the highest expression levels of *CsPOD* (Figure 5B) and *CsAPX* (Figure 5D) at 24 h of NaCl treatment and 48 h of 4 °C treatment than those of non-treated plants (6.08 and 2.93-fold higher, respectively). These results suggest that melatonin could mediate the expression of key genes involved in the biosynthesis of antioxidant enzymes in tea plants under abiotic stress.

## 3. Discussion

Abiotic stresses markedly inhibit plant growth via different mechanisms and result in a decrease of crop yield. In recent years, exogenous substances have been widely used to improve plant stress resistance and crop yield, among these, exogenous melatonin has emerged as a research focus in plant science [38]. Previous studies have shown that exogenous melatonin enhances abiotic stress tolerance in some plant species including *Cynodon dactylon* and *Glycine max* [5,27]. Nevertheless, the mechanisms involved in melatonin-mediated tolerance to abiotic stress in tea plants still remain unknown. Thus, we examined the effects of melatonin on photosynthetic, ROS accumulation and antioxidant defense systems in tea plants under cold, high salt and drought stress. The present study indicated that the application of melatonin plays a protective role in tea plants against cold, high salt and drought stress.

The effect of exogenously applied melatonin ranges from a significant amelioration to being ineffective or even toxic [22]. Exogenous melatonin promoted rooting at low concentration but inhibited the growth of tissue culture at high concentration [39]. There were different concentrations applied to different plant species and organs. Our previous study indicated that 100 μM melatonin was the most effective concentration for tea leaves [40]. In the present study, we analyzed the effects of 100 μM melatonin on tea plants under abiotic stress. To examine the photosynthesis of tea leaves when exposed to cold, salt and drought stress, we monitored the time-course of changes in values of F_v_/F_m_. F_v_/F_m_ reflects the maximum photochemical efficiency of PSII and is used to reflect the degree of damage to the photosynthetic apparatus in stress processes [41]. Although abiotic stress significantly decreased F_v_/F_m_, melatonin-treated leaves maintained a relatively higher F_v_/F_m_ compared with the non-melatonin treated plants (Figure 1). This is consistent with the finding reported in tomato under cold-induced stress [42]. It is speculated that melatonin improves photosynthetic efficiency by improving the efficiency of photosystem II in plants [30]. These results suggest that exogenous melatonin enhanced abiotic stress resistance through increasing the efficiency of photosystem in tea plants.

Abiotic stress-induced decrease of PSII activity results in accumulation of ROS such as H_2_O_2_ and O_2_^−^. Although low levels of ROS are indispensable in a plant, excessive accumulations have been proven to causes lipid peroxidation, enzyme inactivation and DNA damage [15]. Previous studies have exhibited that exogenous melatonin can decrease ROS in plants exposed to cold stress [43], salinity stress [44], drought stress [45], and during senescence [30]. In our study, the H_2_O_2_ and O_2_^−^ content was remarkably induced by the treatment of 4 °C, NaCl and drought, but the application of exogenous melatonin alleviated abiotic stress-triggered ROS accumulation (Table 1). Since a decreased ROS level can alleviate oxidative injury of cell membranes in melatonin-treated tea plants, we speculated that the content of MDA in these plants was lower compared with non-melatonin-treated tea plants under abiotic stress. To validate this, we measured the level of MDA in tea leaves. The results show that a lower level of MDA was observed in melatonin-treated seedlings under stress conditions (Figure 2). MDA content is generally considered as a reliable indicator of oxidative damage reflecting cell membrane stability [11]. This is consistent with the finding reported in cucumber under salt and cold stress [46,47]. Taken together, these results show that melatonin helps to reduce membrane damage caused by over-accumulation of ROS. 

To cope with oxidative stress induced by adverse conditions, plants have evolved an effective antioxidant defense system, including enzymatic and non-enzymatic antioxidants. It was reported that under oxidative stress, ROS generation increases antioxidant enzymes activities in plants [43]. Many studies have confirmed that melatonin enhances the activity of antioxidant enzymes under abiotic stress [5,32,33,48]. Consistently, in this study, oxidative stress dramatically activated the activities of SOD, CAT, POD and APX (Figure 3). Interestingly, melatonin enhanced the activities of the antioxidant enzymes (SOD, CAT, POD and APX) further at different time points after 4 °C, NaCl and drought treatment. We also analyzed the effects of exogenous melatonin on the expression of antioxidant enzyme biosynthesis genes (*CsSOD*, *CsPOD*, *CsCAT* and *CsAPX*) (Figure 5). Exogenous melatonin significantly increased the transcription levels of *CsSOD*, *CsPOD*, *CsCAT* and *CsAPX*. Other studies have shown that melatonin could regulate the corresponding genes of antioxidant enzymes in various species [32,49]. Consistent with these studies, these findings confirm that melatonin stimulates the activities of the main antioxidant enzymes under abiotic stress by increasing the expression level of the corresponding genes, thereby improving the tea plant stress resistance. In addition, GSH and ASA, two important non-enzymatic antioxidants in the ASA-GSH cycle, are vital antioxidants against oxidative stress by scavenging ROS in plants [46,50,51]. In a plant cell, O_2_^−^ can be rapidly converted to H_2_O_2_ by SOD, while H_2_O_2_ can be scavenged by an ASA and/or a GSH regenerating cycle and CAT [10,52]. In the present study, GSH and ASA contents were markedly induced in melatonin-treated treatments under abiotic stress (Figure 4). Similar studies had been reported previously [5,11,46]. Taken together, the above results suggest that exogenous application of melatonin activated ROS detoxification of antioxidants, including enzymatic antioxidant enzymes (SOD, POD, CAT and APX) and non-enzymatic antioxidants (GSH and ASA) to maintain cellular ROS (H_2_O_2_ and O_2_^−^) at a relatively low level.

In conclusion, this study shows the ameliorative effects of exogenous application of melatonin on abiotic stress to tea plants. The present study provides the first evidence of the protective roles of exogenous melatonin response to multiple abiotic stresses in tea plants. To be specific, melatonin improves abiotic stress tolerance in tea plants, possibly through the enhancement of photosynthesis, the elimination of ROS and the upregulation of some key components of the antioxidant system. Our work also provides a case study that melatonin may have great potential for improving tea plant yield. However, further studies need to be conducted to provide more molecular and genetic evidence to support the mechanisms of melatonin-induced abiotic stress tolerance in tea plants.

## 4. Materials and Methods

### 4.1. Plant Material and Treatments

Two-year-old vegetatively propagated cuttings of tea plants (*C. sinensis cv*. ‘Longjing 43’) were cultivated in a chamber at the Tea Research Institute of Nanjing Agricultural University (Nanjing, China). Tea plants were grown in a growth chamber under 25 °C for 14 h with 200 μmol m^−2^ s^−1^ light intensity at daytime, at 18 °C for 10 h during night time and 70% of relative humidity. All plants were allowed to acclimatize for around 1 month before treatment, and every 60 tea cuttings seedlings were used for each treatment. Hoagland’s nutrient solution was used for fertilization with applications every 6 days, and proper moisture level was maintained by daily watering. 

The tea plants were randomized into eight groups: control condition (CK), control condition plus melatonin (CK + melatonin), cold condition (4 °C), cold condition plus melatonin (4 °C + melatonin), high salt condition (NaCl), high salt condition plus melatonin (NaCl + melatonin), drought condition (Drought) and drought condition plus melatonin (drought + melatonin). For the control, plants were treated with 25 °C and irrigated with deionized water until the experiment ended. For the melatonin treatment, tea plants were pretreated with 100 μM melatonin (Sigma-Aldrich, St. Louis, MO, USA) solution for 7 days (once a day). This concentration was selected based on our preliminary tests and all treatments were sprayed as a volume that saturated the canopy (approximately 100 mL per plot). After pre-treatment, the plants were subjected to cold stress (4 °C), high salt (300 mM NaCl) and drought stress (200 g·L^−1^ PEG) [53,54]. Leaf samples were harvested at 0, 12, 24 and 48 h after abiotic stress treatment from each group, immediately frozen in liquid nitrogen and stored at −80 °C for subsequent analysis of physiological indicators and gene expression. Three independent replicates were performed in all experiments.

### 4.2. Determination of Maximum Photochemical Efficiency of PSII (F_v_/F_m_)

The chlorophyll fluorescence parameters of tea leaves were determined with a portable photosynthesis system (LI6800, LI-COR, Inc. Lincoln, NE, USA). The tea plants were pre-adapted in the dark for 30 min before measurement to ensure sufficient closure of all PSII reaction centers, the 2nd leaves from the top buds of tea plants were used to determine F_v_/F_m_. Minimum fluorescence (F_o_) was measured during the weak measuring pulses, a 0.8-s pulsed light (4000 μmol·m^−2^·s^−1^) was applied to measure the maximum fluorescence (F_m_). F_v_/F_m_ was calculated as (F_m_ − F_0_)/F_m_ [55].

### 4.3. Determination of Malondialdehyde (MDA), Hydrogen Peroxide (H_2_O_2_) and Superoxide Anion (O_2_^−^)

The level of lipid peroxidation in leaves was assessed by measuring the MDA content using 2-thiobarbituric acid as described by Hodges et al. [56].

For the H_2_O_2_ assay, 0.3 g of tea leaf samples were grounded with 3 mL of pre-cooled HClO_4_ (1 M) using a pre-chilled mortar and pestle, and the homogenate was centrifuged (TGL-10C, Anting, Shanghai, China) at 6000× *g* for 5 min at 4 °C [57]. The pH of the supernatant was adjusted to 6.0 with 4 M KOH and centrifuged at 12,000× *g* for 5 min at 4 °C. Afterward, the resulting supernatant was passed through an AG1x8 prepacked column (Bio-Rad, Hercules, CA, USA) and H_2_O_2_ was eluted with 4 mL double-distilled H_2_O. The sample (800 μL) was mixed with 400 μL reaction buffer containing 4 mM 2,2′-azino-di (3-ethylbenzthiazoline-6-sulfonic acid) and 100 mM potassium acetate at pH 4.4, 400 μL deionized water and 0.25 U of horseradish peroxidase (HRP). H_2_O_2_ content was measured at 412 nm using UV spectrophotometer (UV-5800H, Yuanxi, Shanghai, China).

O_2_^−^ production was estimated as described by Elstner and Heupel [58] with slight modification. The leaf sample (0.5 g) was homogenized with 3 mL of 65 mM potassium phosphate buffer (pH 7.8) and centrifuged at 8000× *g* for 10 min. Then, 1 mL of the supernatant was mixed with 0.9 mL of 65 mM phosphate buffer (pH 7.8) and 0.1 mL of 10 mM hydroxylamine hydrochloride. The mixture was incubated at 25 °C for 20 min. Then, 0.375 mL of 17 mM sulfanilamide and 0.375 mL of 7 mM α-amino-phenylsulfonic were added to the mixture for another 20 min of incubation at 30 °C. Next, ethylether in the same volume was added and centrifuged at 2000× *g* for 5 min. The absorption of the reaction mixture was measured at 530 nm. Sodium nitrite was used as a standard solution to calculate the production rate of superoxide anion.

### 4.4. Extraction and Assay of the Antioxidant Enzymes

Fresh leaves (0.5 g) were grounded on ice with 0.5 g quartz sands and 5.0 mL of 50 mM precooled phosphate buffer (pH 7.5) containing 0.1 mM ethylene diamine tetraacetic acid (EDTA) and 5.0% (*w*/*v*) polyvinylpyrrolidone (PVP). The homogenate was centrifuged at 4 °C for 15 min at 12,000× *g* [59]. The resulting supernatant was used for the analysis of superoxide dismutase (SOD), catalase (CAT), peroxidase (POD) and ascorbate peroxidase (APX). All the above antioxidant enzymes were determined by using the reagent kit (Jiancheng Bioengineering Institute, Nanjing, China) according to the manufacturer’s instruction.

### 4.5. Extraction and Analysis of Glutathione (GSH) and Ascorbate (ASA)

The content of GSH was measured following the procedure described by Griffith [60]. 0.1 g of leaf tissue was homogenized in 2 mL of 5% sulfosalicylic acid at 4 °C. The homogenate was centrifuged at 12,000× *g* for 10 min. For measurement of GSH, the resulting supernatant was mixed with 20 μL of 10 mM NADPH, 80 μL of 12.5 mM 5,5′-dithio-bis-(2-nitrobenzoic acid) (DTNB), and 700 μL of 50 mM phosphate buffer (pH 7.5) containing 2.5 mM EDTA. Then, 20 μL of GR (50 U/mL) was added to the supernatant and the increase in absorbance at 412 nm was used for calculating the content of GSH.

The content of AsA was assayed by the method of Logan et al. [61]. The crude extract of AsA was obtained by homogenizing 0.1 g fresh leaves in 6% (v/v) cold HClO_4_. The crude extract was centrifuged at 12,000× *g* for 10 min at 4 °C, the resulting supernatant was collected for further analysis. AsA was assayed by determining the absorbance difference of the supernatant at 265 nm in 200 mM sodium acetate buffer (pH 5.6) before and after 15-min incubation with 1.5 units of AsA oxidase.

### 4.6. Total RNA Extraction cDNA Synthesis and qRT-PCR Assay

Total RNA was isolated from tea plants using the Quick RNA Isolation Kit (Huayueyang, Beijing, China). The concentration and purity of RNA were measured using a NanoDrop^TM^ 2000 spectrophotometer (Thermo Scientific, Wilmington, DE, USA). The cDNA of tea leaves was synthesized using a PrimeScript^TM^ RT reagent kit (TaKaRa, Dalian, China), following the manufacturer’s instructions. Quantitative real-time PCR (qRT-PCR) was performed on an IQ5 multicolor real-time PCR detection system (Bio-Rad, Hercules, CA, USA). The reaction program was set as follows: 95 °C for 30 s followed by 40 cycles at 95 °C for 5 s and 55 °C for 25 s. The reaction volume was 20 μL, which contained 2 μL diluted cDNA strand, 7.2 μL ddH_2_O, 10 μL SYBR^®^ Premix Ex Taq (TaKaRa, Dalian, China), and 0.4 μL each primer. The *C. sinensis β-actin* gene was selected as an internal control gene. These qRT-PCR experiments were repeated three times, based on three separate RNA extracts from three samples. Relative gene expression levels were calculated using the 2^−ΔΔCT^ method [62]. Detection primers were designed using Primer Premier 5.0 version (Biosoft International, Palo Alto, CA, USA). The primer sequences used in this study are listed in Table S1 (Supplementary Materials).

### 4.7. Statistical Analysis

The experiment had a completely randomized design. Each value was expressed as the mean ± SE of the three independent experiments. All data were analyzed using SPSS 20.0 (Windows, Chicago, IL, USA). Significance was determined by Duncan’s test and ANOVA. Different letters in figures indicate significant differences at *P* < 0.05.

## Figures and Tables

**Table 1:** Effect of exogenous melatonin on H_2_O_2_ and O_2_^−^ levels in tea leaves on the designated times.

Treatments	H_2_O_2_ Content (μmol/g FW)				O_2_^−^ Content (μmol/g FW)			
	0 h	12 h	24 h	48 h	0 h	12 h	24 h	48 h
CK	0.321 ± 0.01 ^m^	0.473 ± 0.007 ^j^	0.445 ± 0.008 ^jk^	0.438 ± 0.006 ^jk^	0.977 ± 0.009 ^f^	0.863 ± 0.004 ^g^	0.863 ± 0.021 ^g^	0.816 ± 0.011 ^g^
CK + MT	0.348 ± 0.019 ^lm^	0.468 ± 0.002 ^j^	0.347 ± 0.005 ^lm^	0.444 ± 0.021 ^jk^	0.99 ± 0.01 ^f^	0.831 ± 0.065 ^g^	0.87 ± 0.009 ^g^	0.977 ± 0.109 ^f^
4 °C	0.355 ± 0.008 ^lm^	0.445 ± 0.014 ^jk^	1.339 ± 0.02 ^b^	1.281 ± 0.001 ^c^	0.967 ± 0.016 ^f^	1.579 ± 0.007 ^b^	1.159 ± 0.016 ^e^	1.703 ± 0.042 ^a^
4 °C + MT	0.316 ± 0.004 ^m^	0.402 ± 0.019 ^kl^	0.824 ± 0.012 ^fg^	1.054 ± 0.076 ^d^	0.967 ± 0.044 ^f^	1.334 ± 0.039 ^c^	0.844 ± 0.019 ^g^	0.953 ± 0.002 ^f^
NaCl	0.347 ± 0.014 ^lm^	0.652 ± 0.032 ^h^	1.225 ± 0.025 ^c^	1.531 ± 0.019 ^a^	0.867 ± 0.008 ^g^	0.866 ± 0.007 ^g^	0.871 ± 0.009 ^g^	0.994 ± 0.009 ^f^
NaCl + MT	0.323 ± 0.023 ^m^	0.58 ± 0.016 ^i^	0.577 ± 0.004 ^i^	0.874 ± 0.007 ^f^	0.978 ± 0.029 ^f^	0.707 ± 0.014 ^h^	0.729 ± 0.012 ^h^	0.827 ± 0.011 ^g^
Drought	0.319 ± 0.008 ^m^	0.376 ± 0.015 ^lm^	0.787 ± 0.022 ^g^	0.986 ± 0.007 ^e^	0.974 ± 0.007 ^f^	0.517 ± 0.006 ^b^	1.361 ± 0.006 ^c^	1.283 ± 0.02 ^cd^
Drought + MT	0.363 ± 0.012 ^lm^	0.324 ± 0.015 ^m^	0.575 ± 0.009 ^i^	0.83 ± 0.026 ^fg^	1.022 ± 0.04 ^f^	1.174 ± 0.021 ^e^	1.209 ± 0.014 ^de^	0.857 ± 0.027 ^g^

The tea plants were subjected to control (CK), cold stress (4 °C), high salt (300 mM NaCl) and drought stress (200 g·L^−1^ PEG) with different treatments (CK and 100 μM melatonin). Data represent means ± SD of three replicate samples. Different letters indicate significant differences according to Duncan’s multiple range test (*P* < 0.05). MT, melatonin.

## Supplementary Materials

The following are available online, Table S1: Primers used for gene expression analysis.

## References

[1] Chen Y., Yu M., Xu J., Chen X., Shi J. (2009). Differentiation of eight tea (Camellia sinensis) cultivars in China by elemental fingerprint of their leaves. J. Sci. Food Agric..

[2] Wang L., Cao H., Qian W., Yao L., Hao X., Li N., Yang Y., Wang X. (2017). Identification of a novel bZIP transcription factor in Camellia sinensis as a negative regulator of freezing tolerance in transgenic arabidopsis. Ann. Bot..

[3] Li X., Ahammed G.J., Li Z., Zhang L., Wei J., Yan P., Zhang L., Han W. (2018). Freezing stress deteriorates tea quality of new flush by inducing photosynthetic inhibition and oxidative stress in mature leaves. Sci. Hortic..

[4] Xiong L., Schumaker K.S., Zhu J. (2002). Cell Signaling during Cold, Drought, and Salt Stress. Plant Cell.

[5] Shi H., Jiang C., Ye T., Tan D.X., Reiter R.J., Zhang H., Liu R., Chan Z. (2015). Comparative physiological, metabolomic, and transcriptomic analyses reveal mechanisms of improved abiotic stress resistance in bermudagrass [*Cynodon dactylon* (L). Pers.] by exogenous melatonin. J. Exp. Bot..

[6] Zhou L., Xu H., Mischke S., Meinhardt L.W., Zhang D., Zhu X., Li X., Fang W. (2014). Exogenous abscisic acid significantly affects proteome in tea plant (*Camellia sinensis*) exposed to drought stress. Hortic. Res..

[7] Hou Y.D., Guo Z.F., Yi Y., Li H.N., Li H.G., Chen L.J., Ma H., Zhang L., Lin J.W., Zhong M. (2010). Effects of cold acclimation and exogenous pytohormone abscisic acid treatment on physiological indicators of winterness wheat. J. Plant Sci..

[8] Meloni D.A., Oliva M.A., Martinez C.A., Cambraia J. (2003). Photosynthesis and activity of superoxide dismutase, peroxidase and glutathione reductase in cotton under salt stress. Environ. Exp. Bot..

[9] Dahal K., Kane K., Gadapati W., Webb E., Savitch L.V., Singh J., Sharma P., Sarhan F., Longstaffe F.J., Grodzinski B. (2012). The effects of phenotypic plasticity on photosynthetic performance in winter rye, winter wheat and Brassica napus. Physiol. Plant..

[10] Li H., Chang J., Chen H., Wang Z., Gu X., Wei C., Zhang Y., Ma J., Yang J., Zhang X. (2017). Exogenous Melatonin Confers Salt Stress Tolerance to Watermelon by Improving Photosynthesis and Redox Homeostasis. Front. Plant Sci..

[11] Chen Y., Mao J., Sun L., Huang B., Ding C., Gu Y., Liao J., Hu C., Zhang Z., Yuan S. (2018). Exogenous melatonin enhances salt stress tolerance in maize seedlings by improving antioxidant and photosynthetic capacity. Physiol. Plant..

[12] Mittler R., Vanderauwera S., Gollery M., Van Breusegem F. (2004). Reactive oxygen gene network of plants. Trends Plant Sci..

[13] Woo H.R., Kim J.H., Nam H.G., Lim P.O. (2004). The Delayed Leaf Senescence Mutants of Arabidopsis, ore1, ore3, and ore9 are Tolerant to Oxidative Stress. Plant Cell Physiol..

[14] Allakhverdiev S.I., Kreslavski V.D., Klimov V.V., Los D.A., Carpentier R., Mohanty P. (2008). Heat stress: An overview of molecular responses in photosynthesis. Photosynth. Res..

[15] Marta B., Szafranska K., Posmyk M.M. (2016). Exogenous Melatonin Improves Antioxidant Defense in Cucumber Seeds (*Cucumis sativus* L.) Germinated under Chilling Stress. Front. Plant Sci..

[16] Ding F., Wang G., Zhang S. (2018). Exogenous Melatonin Mitigates Methyl Viologen-Triggered Oxidative Stress in Poplar Leaf. Molecules.

[17] Calvo J.R., Gonzã l.-Y.C., Maldonado M.D. (2013). The role of melatonin in the cells of the innate immunity: A review. J. Pineal Res..

[18] Hattori A., Migitaka H., Iigo M., Itoh M., Yamamoto K., Ohtanikaneko R., Hara M., Suzuki T., Reiter R.J. (1995). Identification of melatonin in plants and its effects on plasma melatonin levels and binding to melatonin receptors in vertebrates. Biochem. Mol. Biol. Int..

[19] Dubbels R., Reiter R.J., Klenke E., Goebel A., Schnakenberg E., Ehlers C., Schiwara H.W., Schloot W. (1995). Melatonin in edible plants identified by radioimmunoassay and by high performance liquid chromatography-mass spectrometry. J. Pineal Res..

[20] Manchester L.C., Cotomontes A., Boga J.A., Andersen L.P.H., Zhou Z., Galano A., Vriend J., Tan D.X., Reiter R.J. (2015). Melatonin: An ancient molecule that makes oxygen metabolically tolerable. J. Pineal Res..

[21] Wang P., Sun X., Li C., Wei Z., Liang D., Ma F. (2013). Long-term exogenous application of melatonin delays drought-induced leaf senescence in apple. J. Pineal Res..

[22] Zhang N., Sun Q., Zhang H., Cao Y., Weeda S., Ren S., Guo Y. (2015). Roles of melatonin in abiotic stress resistance in plants. J. Exp. Bot..

[23] Gao W., Zhang Y., Feng Z., Bai Q., He J., Wang Y. (2018). Effects of Melatonin on Antioxidant Capacity in Naked Oat Seedlings under Drought Stress. Molecules.

[24] Nawaz M.A., Huang Y., Bie Z., Waqar A., Reiter R.J., Niu M., Saba H. (2016). Melatonin: Current Status and Future Perspectives in Plant Science. Front. Plant Sci..

[25] Arnao M.B., Hernández-Ruiz J. (2014). Melatonin: Plant growth regulator and/or biostimulator during stress?. Trends Plant Sci..

[26] Arnao M.B., Hernández-Ruiz J. (2017). Melatonin and its relationship to plant hormones. Ann. Bot..

[27] Wei W., Li Q.T., Chu Y.N., Reiter R.J., Yu X.M., Zhu D.H., Zhang W.K., Ma B., Lin Q., Zhang J. (2015). Melatonin enhances plant growth and abiotic stress tolerance in soybean plants. J. Exp. Bot..

[28] Annia G., Dun Xian T., Reiter R.J. (2013). On the free radical scavenging activities of melatonin’s metabolites, AFMK and AMK. J. Pineal Res..

[29] Tan D.X., Manchester L.C., Mascio P.D., Martinez G.R., Prado F.M., Reiter R.J. (2007). Novel rhythms of N1-acetyl-N2-formyl-5-methoxykynuramine and its precursor melatonin in water hyacinth: importance for phytoremediation. FASEB J..

[30] Wang P., Yin L., Liang D., Li C., Ma F., Yue Z. (2012). Delayed senescence of apple leaves by exogenous melatonin treatment: toward regulating the ascorbate–glutathione cycle. J. Pineal Res..

[31] Zhang H., Zhang Y. (2014). Melatonin: A well-documented antioxidant with conditional pro-oxidant actions. J. Pineal Res..

[32] Zhang H., Zhang N., Yang R., Wang L., Sun Q., Li D., Cao Y., Weeda S., Zhao B., Ren S. (2014). Melatonin promotes seed germination under high salinity by regulating antioxidant systems, ABA and GA4 interaction in cucumber (*Cucumis sativus* L.). J. Pineal Res..

[33] Sharif R., Xie C., Zhang H., Arnao M.B., Ali M., Ali Q., Muhammad I., Shalmani A., Nawaz M.A., Chen P. (2018). Melatonin and Its Effects on Plant Systems. Molecules.

[34] Janas K.M., Posmyk M.M. (2013). Melatonin, an underestimated natural substance with great potential for agricultural application. Acta Physiol. Plant..

[35] Kolodziejczyk I., Posmyk M.M. (2016). Melatonin—A new plant biostimulator?. J. Elementol..

[36] Reiter R.J., Tan D.X., Zhou Z., Cruz M.H.C., Fuentes-Broto L., Galano A. (2015). Phytomelatonin: Assisting plants to survive and thrive. Molecules.

[37] Arnao M.B., Hernández-Ruiz J. (2015). Functions of melatonin in plants: A review. J. Pineal Res..

[38] Posmyk M.M., Janas K.M. (2009). Melatonin in plants. Acta Physiol. Plant..

[39] Sarropoulou V., Dimassitheriou K., Therios I., Koukourikoupetridou M. (2012). Melatonin enhances root regeneration, photosynthetic pigments, biomass, total carbohydrates and proline content in the cherry rootstock PHL-C (Prunus avium × Prunus cerasus). Plant Physiol. Biochem..

[40] Li J., Arkorful E., Cheng S., Zhou Q., Li H., Xuan C., Kang S., Li X. (2018). Alleviation of cold damage by exogenous application of melatonin in vegetatively propagated tea plant (*Camellia sinensis* (L.) O. Kuntze). Sci. Hortic..

[41] Kusaba M., Ito H., Morita R., Iida S., Sato Y., Fujimoto M., Kawasaki S., Tanaka R., Hirochika H., Nishimura M. (2007). Rice NON-YELLOW COLORING1 is involved in light-harvesting complex II and grana degradation during leaf senescence. Plant Cell.

[42] Fei D., Liu B., Zhang S. (2017). Exogenous melatonin ameliorates cold-induced damage in tomato plants. Sci. Hortic..

[43] Fan J., Hu Z., Xie Y., Chan Z., Chen K., Amombo E., Chen L., Fu J. (2015). Alleviation of cold damage to photosystem II and metabolisms by melatonin in Bermudagrass. Front. Plant Sci..

[44] Li C., Wang P., Wei Z., Liang D., Liu C., Yin L., Jia D., Fu M., Ma F. (2012). The mitigation effects of exogenous melatonin on salinity-induced stress in Malus hupehensis. J. Pineal Res..

[45] Zhang N., Zhao B., Zhang H.J., Weeda S., Yang C., Yang Z.C., Ren S., Guo Y.D. (2012). Melatonin promotes water-stress tolerance, lateral root formation, and seed germination in cucumber (*Cucumis sativus* L.). J. Pineal Res..

[46] Zhao H., Ye L., Wang Y., Zhou X., Yang J., Wang J., Cao K., Zou Z. (2016). Melatonin Increases the Chilling Tolerance of Chloroplast in Cucumber Seedlings by Regulating Photosynthetic Electron Flux and the Ascorbate-Glutathione Cycle. Front. Plant Sci..

[47] Wang L.Y., Liu J.L., Wang W.X., Sun Y. (2016). Exogenous melatonin improves growth and photosynthetic capacity of cucumber under salinity-induced stress. Photosynthetica.

[48] Jiang C., Cui Q., Feng K., Xu D., Li C., Zheng Q. (2016). Melatonin improves antioxidant capacity and ion homeostasis and enhances salt tolerance in maize seedlings. Acta Physiol. Plant..

[49] Zhang N., Zhang H.J., Zhao B., Sun Q.Q., Cao Y.Y., Li R., Wu X.X., Weeda S., Li L., Ren S. (2014). The RNA-seq approach to discriminate gene expression profiles in response to melatonin on cucumber lateral root formation. J. Pineal Res..

[50] Nagalakshmi N., Prasad M.N.V. (2001). Responses of glutathione cycle enzymes and glutathione metabolism to copper stress in Scenedesmus bijugatus. Plant Sci..

[51] Kocsy G., Galiba G., Brunold C. (2001). Role of glutathione in adaptation and signalling during chilling and cold acclimation in plants. Physiol. Plant..

[52] Foyer C.H., Noctor G. (2011). Ascorbate and Glutathione: The Heart of the Redox Hub. J. Plant Physiol..

[53] Wu X., Zeng F., Zhang G. (2017). PEG-simulated drought stress and spike in vitro culture are used to study the impact of water stress on barley malt quality. Plant Growth Regul..

[54] Cui Z.H., Bi W.L., Hao X.Y., Xu Y., Li P.M., Walker M.A., Wang Q.C. (2016). Responses ofIn vitro-Grown Plantlets (Vitis vinifera) toGrapevine leafroll-Associated Virus-3and PEG-Induced Drought Stress. Front. Physiol..

[55] Li X., Ahammed G.J., Zhang Y.Q., Zhang G.Q., Sun Z.H., Zhou J., Zhou Y.H., Xia X.J., Yu J.Q., Shi K. (2015). Carbon dioxide enrichment alleviates heat stress by improving cellular redox homeostasis through an ABA-independent process in tomato plants. Plant Biol..

[56] Hodges D.M., Delong J.M., Forney C.F., Prange R.K. (1999). Improving the thiobarbituric acid-reactive-substances assay for estimating lipid peroxidation in plant tissues containing anthocyanin and other interfering compounds. Planta.

[57] Willekens H., Chamnongpol S., Davey M.W., Schraudner M., Langebartels C., Van Montagu M., Inze D., Van Camp W. (1997). Catalase is a sink for H2O2 and is indispensable for stress defence in C3 plants. EMBO J..

[58] Elstner E.F., Heupel A. (1976). Inhibition of nitrite formation from hydroxylammoniumchloride: A simple assay for superoxide dismutase. Anal. Biochem..

[59] Pereira G.J.G., Molina S.M.G., Lea P.J., Azevedo R.A. (2002). Activity of antioxidant enzymes in response to cadmium in Crotalaria juncea. Plant Soil.

[60] Griffith O.W. (1980). Determination of glutathione and glutathione disulfide using glutathione reductase and 2-vinylpyridine. Anal. Biochem..

[61] Logan B.A., Grace S.C., Adams W.W., Demmig-Adams B. (1998). Seasonal differences in xanthophyll cycle characteristics and antioxidantsin Mahonia repens growing in different light environments. Oecologia.

[62] Livak K.J., Schmittgen T.D. (2012). Analysis of relative gene expression data using real-time quantitative PCR and the 2(-Delta Delta C(T)) Method. Methods.

