# Supplementary material for: Exogenous Melatonin Enhances Cold, Salt and Drought Stress Tolerance by Improving Antioxidant Defense in Tea Plant (Camellia sinensis (L.) O. Kuntze)
Source: Molecules. 2019 May 12;24(9):1826. doi: 10.3390/molecules24091826 (PMC6539935; doi:10.3390/molecules24091826)
Supplement: Supplementary file 1 [file molecules-24-01826-s001.pdf]

Table S1 Primers used for gene expression analysis

**Name**	**Forward primer(5'-3')**	**Reverse primer(5'-3')**
CsSOD	GATGACGGAACTGCTTGCTT	ATCAGGGTCTGCATGGACAA
CsPOD	GCCACACTTCGCTTATTCTT	AGCCAGGACTACAACATCTC
CsCAT	CCTGAACGTGTTGTCCATGC	AACCTCGAGGATCCCTCAG
CsAPX	CAGTTCCCGATGATCTCTTATGC	GCAACAATGTCCTTGTCAGTGAG
Csβ-actin	GATTCCGTTGCCCTGAAGTCCT	CCTTGCTCATACGGTCTGCGATA
